# 779 Utilization of Palliative Care in Patients Succumbing to Primary Burn Injury

**DOI:** 10.1093/jbcr/irad045.254

**Published:** 2023-08-29

**Authors:** Alisa Savetamal, Joseph Canner, Karen Gibbs, Elena Graetz, Chin Siang Ong, Eric Schneider

**Affiliations:** Yale New Haven Health, Bridgeport Hospital, Bridgeport, Connecticut; Yale University, New Haven, Connecticut; Yale-New Haven Health/Bridgeport Hospital, Bridgeport, Connecticut; Yale University, New Haven, Connecticut; Yale, New Haven, Connecticut

## Abstract

**Introduction:**

Burn injury is associated with significant morbidity and mortality. Access to palliative care (PC) is variable, and many patients who die after burns do not receive specialized PC consultation. We conducted a retrospective nation-wide study of patients admitted with a primary diagnosis of burn injury who died prior to hospital discharge to identify factors associated with receiving palliative care.

**Methods:**

Data from the HCUP National Inpatient Sample (2016-2019) were queried to identify inpatient deaths among adults ≥18 admitted with a primary ICD-10 burn diagnosis. Differences in demographic and clinical characteristics were assessed between patients who did and did not receive PC using chi-square tests and multivariable Poisson regression on data weighted to represent the national population. Baux scores were calculated by adding age and percentage of body burnt based upon ICD-10 code categorizations; a revised Baux score was calculated for inhalation injury.

**Results:**

Of 98,845 patients admitted to inpatient care from 2016 to 2019 with a primary burn diagnosis, 3250 (3.3%) died prior to discharge. 1390 (42.8%) of patients who died had a PC encounter. Patients who did not receive PC were younger (mean 58.7 vs. 63.9, p=0.0002), had lower Baux scores (mean 104.6 vs. 112.4, p=0.002) and a longer length of stay (LOS) (mean 19.3 vs. 13.3, p=0.008). Controlling for age, sex, Baux score and LOS, White patients had a 35% greater likelihood of receiving PC than Black patients (incidence rate ratio, IRR 1.35, 0.95 CI (1.02,1.79)). All else held constant, patients aged 70-79 had the greatest likelihood (68%, IRR 1.68, 0.95 CI (1.31,2.15)) of receiving PC versus patients < 60 years old. Urban teaching hospitals had the highest percentage of burn patients (92.7% vs. 5.2% non-teaching vs. 2.1% rural) and the highest rate of providing PC (43% vs. 30% non-teaching vs. 20% rural, p=0.446) to patients that died. Insurance type (Medicare, Medicaid, Private, and other) did not correlate with receiving PC (p=0.054). The number of diagnoses, procedures and gender did not differ between deceased patients who did and did not receive PC.

**Conclusions:**

More than half of burn patients who died prior to discharge did not receive a palliative care consultation. Unsurprisingly, older patients were more likely to receive PC. Patients who did not receive PC were more likely to have been younger, Black, have had lower Baux scores or to have been treated in non-urban non-teaching hospitals. The high Baux score of the younger patients (104.6) is associated with significant risk of death; this population might benefit from PC consultation. The discrepancy between White and Black patients in the delivery of PC services is striking. Further study is necessary to identify not only those burn patients most likely to benefit from PC but also the barriers to receiving palliative care in burn injury.

**Applicability of Research to Practice:**

Demonstrates the inconsistency of palliative care use in the burn population

